# Vitamin D Deficiency and Cardiometabolic Risks: A Juxtaposition of Arab Adolescents and Adults

**DOI:** 10.1371/journal.pone.0131315

**Published:** 2015-07-17

**Authors:** Nasser M. Al-Daghri, Yousef Al-Saleh, Naji Aljohani, Majed Alokail, Omar Al-Attas, Abdullah M. Alnaami, Shaun Sabico, Maha Alsulaimani, Mohammed Al-Harbi, Hanan Alfawaz, George P. Chrousos

**Affiliations:** 1 Biomarkers Research Program, Biochemistry Department, College of Science, King Saud University, King Saud University, Riyadh, 11451, Saudi Arabia; 2 Prince Mutaib Chair for Biomarkers of Osteoporosis, Biochemistry Department, College of Science, King Saud University, Riyadh, Saudi Arabia; 3 King Abdulaziz Medical City, College of Medicine, King Saud Bin Abdulaziz University for Health Sciences, Riyadh, 11426, Saudi Arabia; 4 Specialized Diabetes and Endocrine Center, King Fahad Medical City, Faculty of Medicine, King Saud bin Abdulaziz University for Health Sciences, Riyadh, 11525, Saudi Arabia; 5 Biochemistry Department, College of Science, King Saud University, Riyadh, 11451, KSA; 6 Diabetes Centers and Units Administration, Ministry of Health, Riyadh, Saudi Arabia; 7 Department of Food Science and Nutrition, College of Food Science and Agriculture, King Saud University, Riyadh, 11451, Saudi Arabia; 8 First Department of Pediatrics, Athens University Medical School, Athens, 11527, Greece; Children's National Medical Center, Washington, UNITED STATES

## Abstract

The recent exponential surge in vitamin D research reflects the global epidemic of vitamin D deficiency and its potential impact on several chronic diseases in both children and adults. Several subpopulations, including Arab adolescent boys and girls, remain understudied. This study aims to fill this gap. A total of 2225 apparently healthy Saudi adolescents (1187 boys and 1038 girls, aged 13-17 years old) and 830 adults (368 men and 462 women, aged 18-50 years old) were respectively recruited from different public schools and medical practices within Riyadh, Saudi Arabia. Anthropometrics were taken and fasting blood samples withdrawn to examine serum glucose and lipid profile by routine analysis and 25-hydroxyvitamin D by ELISA. Almost half of the girls (47.0%) had vitamin D deficiency as compared to only 19.4% of the boys (*p*<0.001), 36.8% of the adult women and 17.7% of the adult men (*p*<0.001). Furthermore, in boys there were more significant inverse associations between serum 25(OH)vitamin D levels and cardiometabolic indices than girls, while in contrast women had more significant associations than men. Vitamin D deficiency was significantly associated with diabetes mellitus type 2 (DMT2) [OR 3.47 (CI1.26-5.55); *p*<0.05] and pre-DM [OR 2.47 (CI 1.48-4.12); *p*<0.01] in boys. Furthermore, vitamin D insufficiency was significantly associated with abdominal obesity in boys [OR 2.75 (CI 1.1-7.1); *p*<0.05]. These associations for DMT2 and abdominal obesity were not observed in adult males, girls and adult women. Vitamin D deficiency/insufficiency and hyperglycemia is high among Arab adolescents. Vitamin D deficiency is mostly associated with cardiometabolic risk factors in adolescent Arab boys. This indicates a sex- and age-related disadvantage for boys with low vitamin D status and challenges the extra-skeletal protection of vitamin D correction in adolescent females.

## Introduction

In the last few years, there has been an intensified research on the impact of vitamin D in health and disease, thanks to the global epidemic of vitamin D deficiency, which, in turn, has been linked to a plethora of equally prevalent age-related chronic diseases [1]. In the Middle-East region alone, where there is an undeniably ample supply of sunlight all year-round, vitamin D deficiency remains unacceptably high, with rates varying from 30–90% [2]. Risk factors that have been consistently linked to vitamin D deficiency in the MENA (Middle East and North Africa) region, at least in adults, include increasing age, season, female gender and obesity-related diseases, such as insulin resistance, partial or complete metabolic syndrome (MetS) and/or Diabetes mellitus type 2 (DMT2) [3–5]. Furthermore, interventional studies, whether pharmacologic in nature or not, have provided insights that vitamin D correction might translate to a better cardiometabolic profile among Arab adults with underlying chronic conditions, such MetS and/or DMT2 [6–8].

Despite the overwhelming evidence and uniformity of findings with respect to the clinical implications of vitamin D deficiency, several populations, such as children and adolescents, remain understudied [1]. To date, the few studies that were done in the MENA region also pointed to increased prevalence of vitamin D deficiency in the pediatric population [9–11]. Moreover, these studies confirmed several associations between vitamin D levels and select cardiometabolic parameters. However, given that the pediatric population’s physiology and metabolism is different from those of their adult counterparts, it is possible that findings from different populations, might contradict one another, if not producing confusion. In previous studies done in children of a wide age range, there are a number of unknown confounders, such as social and environmental factors, that change continuously as the children develop into full grown adults. These factors need to be controlled as much as possible in order to come up with sound observations that can be replicated in other geographical locations. Such observations will hopefully fill the missing gaps that have slowed down experts in coming up with international recommendations on vitamin D deficiency management in children and adolescents.

In this cross-sectional study, we determined disparities in the prevalence of vitamin D deficiency and its associations to several cardiometabolic parameters and other risk factors among Arab adolescents and adults in Riyadh, Saudi Arabia. The study also aimed to provide new insights into whether risk factors already identified in children or adults hold true for adolescents, and whether these risk factors are gender-specific.

## Materials and Methods

### Subjects

A total of 2225 apparently healthy Saudi adolescents (1187 boys and 1038 girls, aged 13–17 years old) and 830 apparently healthy adults (368 men and 462 women, aged 18–50 years old) were recruited respectively from different public and private schools within Riyadh, Saudi Arabia from February to October, 2013. Ethical approval was granted by the Ethics Committee of the College of Science Research Center, King Saud University, Riyadh, Saudi Arabia. Permission to conduct the study was approved by the Ministry of Education and the principals of the different schools involved. Written informed consents from the parents and the subjects themselves were obtained prior to inclusion. Subjects with acute conditions requiring immediate attention and those with chronic conditions, such as asthma and type 1 diabetes mellitus, were excluded from the study. Adult subjects with acute conditions that require immediate medical attention were also excluded. A pre-designed questionnaire which was piloted and tested for face validity by 3 experts (biochemistry, nutrition and public health) and which included questions on socio-demographic data was administered to all subjects. All participating subjects regardless of eligibility were given credits for their support in the conduct of the study.

### Anthropometrics

Anthropometrics were done at different times according to the break hours for both students and faculty members/school staff. Physical examination was carried out by the attending school physician and nurse to determine whether the participants met the inclusion criteria. Weight and height were recorded to the nearest 0.2 kg and 0.5 cm, respectively, using an appropriate international standard scale (Digital Pearson Scale, ADAM Equipment Inc., USA). Waist, hip and arm circumferences were measured using a non-stretchable tape. Blood pressure was measured twice with a 15 minute interval using a standardized mercury sphygmomanometer. The mean systolic and diastolic blood pressure of two measurements taken 15 min apart was noted.

### Blood Withdrawal and Biochemical Assessments

Eligible subjects (both adolescents and adults) who were able to complete the questionnaire and whose body measurements were taken, were advised to return to the school clinic the following day at a fasting state for blood withdrawal. Blood samples were centrifuged on site and delivered immediately to Prince Mutaib Chair for Biomarkers of Osteoporosis (PMCO), Biochemistry Department, King Saud University in Riyadh, Saudi Arabia, for immediate storage and analysis. Fasting blood glucose and lipid profile were measured routinely. Serum 25-Hydroxyvitamin D was measured using COBAS e-411 automated analyzer (Roche Diagnostics, Indianapolis, IN, USA) in a DEQAS-certified laboratory (PMCO). For serum 25-hydroxyvitamin D assay, the inter- and intra-assay coefficients of variation (CV) were 8.0% and 5.6%, respectively, with a lower detection limit (LOD) of <4ng/ml).

### Cut-offs Used

The various states of vitamin D status were defined as following: deficient (<25 nmol/L); insufficient (25–50 nmol/L); sufficient (50–75 nmol/L) and desirable (>75 nmol/L). Overweight and obesity were based on the gender- and age-specific cut-offs proposed by Cole and colleagues for adolescents [12]. For central obesity, the 90^th^ percentile waist circumference obtained from the adolescent cohort was used as cut-off (>92cm for boys and >86cm for girls). For low HDL-cholesterol, elevated fasting blood glucose and elevated triglycerides, the cut-offs for pediatric subjects from the IDF diagnosis of metabolic syndrome was used [13]. The NCEP-ATP III cut-offs for MetS was used for adults [13].

### Statistical Analysis

Data were analyzed using the SPSS version 16.0 (SPSS, Chicago, IL, USA). Frequencies were presented as percentages (%) with 95% confidence intervals, and continuous variables were presented as mean ± standard error. Chi-Square test was done to compare frequencies and independent Student t-tests were used for comparing normally distributed continuous variables. Bivariate correlation was done to assess associations between variables of interest. Stratified by sex, stepwise linear regression was also done using 25(OH) vitamin D as dependent variable, and the various clinical parameters measured (BMI, waist and hip circumference, systolic and diastolic blood pressure, glucose, triglycerides, HDL-, LDL- and total cholesterol, skin color, sunlight exposure and sunscreen use) as independent variables to determine significant predictors of 25(OH) vitamin D in boys and girls. Multinomial logistic regression analysis was done to determine cardiometabolic risk [odds ratio (OR) (Confidence Interval (CI))] using vitamin D deficiency as independent variable. Significance was set at *p*<0.05.

## Results

### Higher prevalence of cardiometabolic risk factors but lower prevalence of vitamin D deficiency in boys and men than girls and women respectively

Table 1 shows the demographic characteristics of the subjects. In adolescents, there was a significantly higher prevalence of obesity in boys than girls (17.8% versus 12.4%; *p*<0.003). Surprisingly, there was a high prevalence of elevated fasting blood glucose levels equivalent to pre-diabetes mellitus in the adolescent cohort, with boys being significantly higher than girls (20.5% versus 17.7%; *p*<0.001). Low HDL-cholesterol was also more prevalent in boys but, nevertheless, also common in girls (77.5% versus 60.0%; *p*<0.001). There was also a significant difference in the distribution of skin color, with more girls under the “fair” category and more boys under the “light and dark brown” category (*p*<0.001). This may be partly due to the boys’ significantly higher prevalence of sunlight exposure than girls (*p*<0.001), as well as the girls’ significantly higher prevalence of sunscreen use than boys (15.9% versus 5.7%; *p*<0.001). With regards to vitamin D status, almost half of the girls (47.0%) had vitamin D deficiency as compared to only 19.4% in boys (*p*<0.001). In adults, women had a significantly higher prevalence of obesity, abdominal obesity, sunscreen use and vitamin D deficiency than men (*p*-values 0.002, <0.001, <0.001 and <0.001, respectively), while men had a significantly higher prevalence of elevated fasting blood glucose equivalent to DM, elevated triglycerides and sunlight exposure than women (all *p*-values <0.001).

**Table 1:** Demographic Characteristics and Prevalence of Risk Factors in the Subjects Studied.

Total N = 3055	ADOLESCENTS			ADULTS		
	Boys	Girls	P-Value	Men	Women	P-Value
N	1187	1038		368	462	
**BMI Status**						0.002
Overweight	21.9 (19.6, 24.2)	22.4 (19.9, 24.9)	0.003	33.9 (29.1, 38.7)	34.3 (30.0, 38.6)	
Obese	17.8 (15.6, 20.0)	12.4 (10.4, 14.4)		33.6 (28.8, 38.4)	43.4 (38.9, 47.9)	
**Abdominal Obesity**	9.5 (7.8, 11.2)	11.0 (9.1, 12.9)	0.25	13.9 (10.4, 17.4)	41.0 (36.5, 45.5)	<0.001
**Fasting Blood Glucose levels**						<0.001
Normal <5.6mmol/l	75.3 (72.8, 77.8)	80.8 (78.4, 83.2)	<0.001	55.4 (50.3, 60.5)	68.5 (64.3, 72.7)	
Pre-DM (5.6–6.9)mmol/l)	20.5 (18.2, 22.8)	17.7 (15.4, 20.0)		26.9 (22.4, 31.4)	22.8 (19.0, 26.6)	
DM (≥7.0mmol/l)	4.3 (3.2, 5.4)	1.6 (0.8, 2.4)		17.7 (13.8, 21.6)	8.7 (6.1, 11.3)	
**Low HDL-Cholesterol (<1.03mmol/l)**	77.5 (75.1, 79.9)	60.0 (57.0, 63.0)	<0.001	92.1 (89.3, 94.5)	89.0 (86.2, 91.8)	0.13
**Elevated Triglycerides (≥1.7mmol/l)**	16.8 (14.7, 18.9)	14.2 (12.1, 16.3)	0.098	49.2 (44.1, 54.3)	33.0 (28.7, 37.3)	<0.001
**Skin Color**						0.61
Fair	31.4 (28.8, 34.0)	39.1 (36.1, 42.1)	<0.001	32.8 (28.0, 37.6)	28.5 (24.4, 32.6)	
Light Brown	60.2 (57.4, 63.0)	56.4 (53.4, 59.4)		61.8 (56.8, 66.8)	65.4 (61.1, 69.7)	
Dark Brown	7.7 (6.2, 9.2)	4.4 (3.2, 5.6)		5.1 (2.8, 7.4)	5.7 (3.6, 7.8)	
Black	0.7 (0.2, 1.2)	0.1 (0.09, 0.3)		0.3 (-0.26, 0.8)	0.4 (-0.2, 1.0)	
**Sunlight Exposure ≥20minutes**	79.9 (77.6, 82.2)	52.7 (49.7, 55.7)	<0.001	63.4 (58.5, 68.3)	37.5 (33.1, 41.9)	<0.001
**Sunscreen Use**	5.7 (4.4, 7.0)	15.9 (13.7, 18.1)	<0.001	6.2 (3.7, 8.7)	28.6 (24.5, 32.7)	<0.001
**Vitamin D Status**						<0.001
Sufficient (≥50nmol/l)	21.1 (18.8, 23.4)	7.5 (5.9, 9.1)	<0.001	25.6 (21.1, 30.1)	25.5 (21.5, 29.5)	
Insufficient (25–49.9nmol/l)	59.9 (57.1, 62.7)	45.5 (42.5, 48.5)		56.7 (51.6, 61.8)	38.0 (33.6, 42.4)	
Deficient (<25nmol/l)	19.4 (17.2, 21.6)	47.0 (44.0, 50.0)		17.7 (13.8, 21.6)	36.8 (32.4, 41.2)	

Abdominal obesity in children was defined as Boys >92cm (>90^th^ Percentile) and Girls >86cm (>90^th^ Percentile); Abdominal obesity in adults defined as Men >102cm and Women >88cm; Low HDL-cholesterol in children defined as <1.03mmol/L; Low HDL-cholesterol in adults defined as Men <1.03mmol/L and Women <1.29mmol/L; significant at p<0.05.

### Age- and BMI-matched anthropometric and biochemical comparisons among subjects

Table 2 shows the clinical characteristics of subjects. In adolescents, age and BMI were not significantly different between the sexes. Boys had a significantly higher mean WHR, systolic blood pressure, glucose, triglycerides, total cholesterol and 25(OH)vitamin D than girls (all *p*-values <0.001 except glucose, 0.002). Girls had a significantly higher mean waist and hip circumference, diastolic blood pressure and HDL-cholesterol (all *p*-values<0.001). In adults, women had a significantly higher BMI than men (*p*<0.001) as well as waist and hip circumference and borderline with LDL-cholesterol (*p*-values 0.006, <0.001, <0.001, <0.001 and 0.05, respectively). On the other hand, men had significantly higher WHR, systolic blood pressure, glucose and triglycerides than women (all *p*-values <0.001). The rest of the variables in adults, including 25(OH)vitamin D were similar in the two sexes (Table 2).

**Table 2:** Clinical and Metabolic Characteristics of the Subjects Studied.

Total N = 3055	ADOLESCENTS			ADULTS		
	Boys	Girls	P-Value	Men	Women	P-Value
N	1187	1038		368	462	
Age (years)	15.1 ± 0.06	15.1 ± 0.06	0.38	37.0 ± 0.49	36.7 ± 0.37	0.62
BMI (kg/m^2^)	22.9 ± 0.17	23.0 ± 0.16	0.74	28.2 ± 0.37	29.4 ± 0.28	0.006
BMI z-score	-0.006 ± 0.03	0.008 ± 0.03	0.75	—	—	
Waist Circumference (cm)	61.7 ± 0.76	71.5 ± 0.34	<0.001	66.9 ± 1.7	85.7 ± 0.58	<0.001
Hip circumference (cm)	70.2 ± 0.84	93.7 ± 0.39	<0.001	74.2 ± 1.9	108.3 ± 0.62	<0.001
Waist-Hip Ratio	0.88 ± 0.01	0.77 ± 0.01	<0.001	0.92 ± 0.01	0.80 ± 0.01	<0.001
Systolic BP (mmHg)	123.2 ± 0.47	119.3 ± 0.45	<0.001	130.0 ± 0.9	123.6 ± 0.8	<0.001
Diastolic BP (mmHg)	69.0 ± 0.40	71.8 ± 0.40	<0.001	76.3 ± 0.7	75.9 ± 0.6	0.62
Glucose (mmol/l)	5.3 ± 0.04	5.1 ± 0.03	0.002	6.2 ± 0.15	5.7 ± 0.12	0.009
Triglycerides (mmol/l)	1.2 ± 0.02	1.1 ± 0.01	<0.001	2.0 ± 0.08	1.6 ± 0.04	<0.001
Total Cholesterol (mmol/l)	3.8 ± 0.02	3.7 ± 0.03	0.30	4.8 ± 0.06	4.8 ± 0.05	0.38
HDL-Cholesterol (mmol/l)	0.89 ± 0.01	0.97 ± 0.02	0.002	0.78 ± 0.01	0.74 ± 0.13	0.80
LDL-Cholesterol (mmol/l)	2.3 ± 0.02	2.3 ± 0.04	0.20	3.0 ± 0.05	3.4 ± 0.14	0.05
25 (OH) Vitamin D (nmol/l)	39.0 ± 0.56	29.4 ± 0.58	<0.001	40.9 ± 1.1	39.8 ± 1.4	0.53

Data presented as mean ± standard error; significance at p<0.05.

### Vitamin d status is correlated with more cardiovascular risk factors in boys than girls and in women than men

Table 3 shows the associations of 25(OH)vitamin D on the parameters included in the present study. In adolescents, circulating 25(OH)vitamin D in boys had significant inverse associations with age, BMI, blood pressure, glucose and triglycerides (*p*-values<0.01), with positive associations with HDL cholesterol and sunlight exposure. In girls, however, 25(OH)vitamin D was only inversely but significantly associated with hip circumference and skin color, and positively and significantly associated with HDL-cholesterol and sunlight exposure. In contrast, 25(OH)vitamin D was positively associated with age in both men and women, with 25(OH)vitamin D in women having significant positive associations with glucose and all lipids. In men, BMI had the same inverse association with 25(OH)vitamin D and positive association with HDL-cholesterol. The rest of the associations were not significant (Table 3).

**Table 3:** Associations of Select Variables to 25(OH)Vitamin D.

Total N = 3055	ADOLESCENTS		ADULTS	
	Boys	Girls	Men	Women
N	1187	1038	368	462
Age (years)	-0.18**	-0.04	0.19**	0.20**
BMI (kg/m^2^)	-0.17**	-0.06	-0.13*	0.03
Waist Circumference (cm)	0.004	-0.06	-0.11	0.06
Hip circumference (cm)	-0.001	-0.07*	-0.07	0.05
Systolic BP (mmHg)	-0.10**	0.02	0.04	0.06
Diastolic BP (mmHg)	-0.09**	0.05	0.06	0.03
Glucose (mmol/l)	-0.10**	0.02	-0.08	0.13**
Triglycerides (mmol/l)	-0.08**	0.006	-0.01	0.14**
Total Cholesterol (mmol/l)	-0.03	0.06	-0.01	0.22**
HDL-Cholesterol (mmol/l)	0.08*	0.10**	0.13*	0.11*
LDL-Cholesterol (mmol/l)	-0.02	0.05	-0.001	0.15**
Skin Color	-0.04	-0.07*	0.005	0.06
Sunlight Exposure ≥ 20 minutes	0.08**	0.07*	-0.01	0.02
Sunscreen Use	0.01	-0.03	0.06	0.006

Data presented as coefficient (R)

* denotes significance at 0.05 level

** denotes significance at 0.01 level.

### Significant predictors of 25(oh) vitamin d in adolescents and adults

To further confirm associations independent of known confounders, stepwise linear regression was done and shown in Table 4. In boys, BMI and age were the significant predictors of 25(OH)vitamin D, predicting a combined 6% (R = 0.25, adjusted R^2^ = 0.07; *p*<0.001) of the variance perceived in 25(OH)vitamin D levels. HDL-cholesterol was the significant predictor in girls (R = 0.09, adjusted R^2^ = 0.01; *p* = 0.014). Significant predictors for men included age, WHR and HDL-cholesterol (R = 0.31, adjusted R^2^ = 0.09; *p* = 0.0001). Significant predictors for women included age, total cholesterol and LDL-cholesterol (R = 0.31, adjusted R^2^ = 0.1; *p*<0.001).

**Table 4:** Stepwise Regression Analysis using 25(OH) Vitamin D as Dependent Variable.

Model	R	Adjusted R^2^	Standardized β	P-Value/s
**Boys**				
BMI	0.228	0.05	-0.228	<0.001
BMI; Age	0.252	0.06	-0.206; -0.108	<0.001
**Girls**				
HDL	0.086	0.006	0.086	0.014
**Men**				
Age	0.22	0.05	0.22	0.003
Age and WHR	0.27	0.07	0.21; 0.15	0.003; 0.04
Age, WHR, HDL	0.31	0.09	0.24; 0.16; 0.15	0.001; 0.032; 0.039
**Women**				
Age	0.24	0.06	0.24	<0.001
Age and TC	0.29	0.08	0.20; 0.17	<0.001; 0.001
Age; TC; LDL	0.31	0.10	0.19; 0.20; -0.11	<0.001; <0.001; 0.034

Independent variables entered: age, BMI, waist-hip ratio, systolic and diastolic blood pressure, glucose, triglycerides, total (TC), HDL- and LDL-cholesterol, skin color, sunlight exposure ≥20 minutes, sunscreen use. Significant at *p*<0.05.

### 25(oh)vitamin d deficiency is associated with increased odds for elevated glucose and abdominal obesity only in boys

Table 5 shows that vitamin D deficiency is significantly associated with DM [OR 3.47 (CI1.26–5.55); *p*<0.05] and pre-DM in boys [OR 2.47 (CI 1.48–4.12); *p*<0.01]. Furthermore, vitamin D insufficiency is significantly associated with abdominal obesity in boys [OR 2.75 (CI 1.1–7.1); *p*<0.05]. The same risk assessment was done in girls, men and women and found no significance (Table 5).

**Table 5:** Multinomial Logistic Regression Analysis using Elevated Glucose and Abdominal Obesity as Dependent Variables and vitamin D status as Independent Variable.

	ADOLESCENTS		ADULTS	
Dependent Variables	Boys	Girls	Men	Women
**DM (>7mmol/L)** ^**a**^				
Vitamin D Deficiency	3.47 (1.26–5.55)*	0.18 (0.04–0.93)	1.81 (0.67–4.89)	0.32 (0.10–1.01)
Vitamin D Insufficiency	1.04 (0.40–2.75)	0.16 (0.04–0.71)	0.88 (0.40–1.96)	1.67 (0.71–3.99)
Vitamin D Sufficiency	1.0	1.0	1.0	1.0
**Pre-DM (5.6–6.9) mmol/L)** ^**a**^				
Vitamin D Deficiency	2.47 (1.48–4.12)**	1.67 (0.76–3.76)	1.27 (0.55–2.92)	0.67 (0.35–1.27)
Vitamin D Insufficiency	1.26 (0.81–1.94)	1.28 (0.58–2.85)	0.66 (0.34–1.27)	1.22 (0.67–2.24)
Vitamin D Sufficiency	1.0	1.0	1.0	1.0
**Abdominal Obesity** ^**b**^				
Vitamin D Deficiency	1.0 (0.32–3.10)	1.31 (0.38–4.45)	1.94 (0.59–6.41)	0.58 (0.27–1.23)
Vitamin D Insufficiency	2.75 (1.1–7.1)*	1.27 (0.36–4.42)	1.25 (0.47–3.33)	0.98 (0.48–2.0)
Vitamin D Sufficiency	1.0	1.0	1.0	1.0

^a^-covariates entered: age, BMI, total cholesterol, triglycerides, HDL-& LDL-cholesterol

^b^-covariates entered: age, BMI, glucose, total cholesterol, triglycerides, HDL-& LDL-cholesterol

*denotes significance at 0.05 level

**denotes significance at 0.01 level

## Discussion

As there are already many cross-sectional and interventional studies on the associations of 25(OH)vitamin D with metabolic parameters in Arab adults [3–7, 14], the present discussion is focused more on the findings in the adolescent cohort. There are several findings in this study that have not been reported elsewhere. First, while indeed it has been established that vitamin D deficiency is generally more common in females and more so in this region, because of both geographical and cultural reasons, including the significantly lesser intake of vitamin D supplementation than males observed in this study [2, 3, 11], the clinical impact of vitamin D deficiency is still more apparent in adolescent males than females. This is in spite of the fact that Arab school boys aged 13–17 have a significantly higher mean vitamin D level, are more physically active outdoors [15], are more likely to receive multivitamins, and are longer exposed to sunlight than school girls of the same age.

The significant associations of vitamin D status to anthropometric, glucose and lipid parameters we previously observed in a much younger cohort are now confirmed mostly among adolescent males rather than females [11]. One apparent explanation for this is the age difference, despite both cohorts falling under the “pediatric” category. In our previous study [11], the mean age [boys = 12.4±3.7; girls = 11.6±3.7] and BMI [boys = 19.8±5.7; girls = 18.9±4.3] of the subjects studied were much younger and smaller respectively than the present one. The sample size [N = 300 versus N = 2225] and setting [Primary Care Center versus School] may also explain the partial inconsistency of the results. The present study is more similar to the study of Kelishadi and colleagues in terms of sample size, setting, and mean age of population studied [10]. However, there are some striking differences between the previous study and the present one. First is that most significant associations of vitamin D status to cardiometabolic parameters are limited to adolescent boys, not girls. Second, this study did not confirm an association between vitamin D status and BMI in females, which we previously observed in both younger children and adults [11, 14].

The lack of association between vitamin D status and anthropometric variables in adolescent girls confirms findings of the study of Jang and colleagues among Korean school girls [15]. It also reaffirms the advantage of girls over boys in CVD risk with approximately 10-year delay, despite them being more insulin-resistant [16, 17]. In the US, the determinant of cardiovascular risk and insulin sensitivity among obese adolescent girls was the ratio of parathyroid hormone to vitamin D, and not vitamin D alone [18]. Several observations also showed lack of significant associations between vitamin D status and cardiometabolic risk factors in Turkish children and adolescents when split by BMI, and insulin resistance among high school students [19, 20]. Other studies confirmed associations between vitamin D status and insulin sensitivity only among obese adolescents [21].

The lack of consistency in the relations of vitamin D status to cardiovascular risk factors among adolescents raises several issues. First is the issue of confounding factors which significantly influence vitamin D status, that were not uniformly addressed in several studies previously mentioned, such as season [22], ethnicity [23], and geography [24]. Second, the population of interest, adolescents, remains understudied, especially in interventional studies and clinical trials. Recent studies [25], including this, support a lack of association between glycemic parameters and vitamin D deficiency. As such, no definitive conclusions can be drawn thus far. Third, there is a growing counter evidence on the lack of strong beneficial metabolic (not bone-related) effects of vitamin D correction in both the general pediatric population [26], and even more in adults at risk for various chronic diseases [27, 28]. Lastly, this study supports our previous observations in adults on sexual dimorphism in the modulation of different proteins associated with various pathways, including vitamin D function, where activation of 1α25(OH) vitamin D signaling is more pronounced in males than females [29].

Still, the high prevalence of vitamin D deficiency in the Arab adolescent population cannot be ignored, and while vitamin D status does not seem to exert any effect in the glycemic status of the cohort studied, larger studies, both observational and interventional, need to be conducted. Some incidental findings in the present study are worth mentioning. There is for instance, a high prevalence of pre-diabetes in the adolescent Arab population and this was more common among boys than girls, suggesting that pre-diabetes and insulin resistance are influenced by gender even among children [30].

Finally, the established cardioprotective effect of estrogen, which is inherent in premenopausal females [31], including adolescent girls, is concealed by the overwhelmingly high prevalence of another known cardiometabolic risk factor, which is vitamin D deficiency [32–34]. It is worthy to note that the wearing of the veil itself, while not noted in the present study, is a known risk factor for vitamin D deficiency in Islamic and Middle Eastern women [2, 35]. Saudi females, in particular, intentionally avoid sunlight exposure for aesthetic purposes (e.g., fair complexion) [36]. This cardiometabolic advantage in the females of this region, as is true for all women, eventually fades with age and menopause [37].

The authors acknowledge several limitations. Given that the study is cross-sectional, findings cannot report causality. Several important markers, such as insulin and HOMA-IR, were not included in the analysis and might have provided added information on the influence of vitamin D on these indices. Variables such as outdoor physical activity were not included in the questionnaire and skin color was based on self-assessment. Statistically significant differences in cardiometabolic indices (e.g. HDL-cholesterol) between groups do not hold much clinical relevance aside from groups having very low values. Nevertheless, the large sample size utilized for this cohort, and the controlled environment in which the study was conducted, makes the findings generalizable for the Arab adolescent population.

In conclusion, the present study highlighted the high prevalence of vitamin D deficiency in the Arab adolescent population, which is more common in girls, but more associated with cardiometabolic risk factors in boys, indicating a sex- and age- disadvantage for boys with low vitamin D status. Hyperglycemia was also common in the Arab adolescent population and was not associated with vitamin D, suggesting that the effects of vitamin D correction maybe most beneficial in a select group of at-risk children and adolescents (e.g., obese adolescent boys) when it comes to glycemia, but this needs to be addressed in large scale interventional studies.
